# Trauma in Health Care Settings: A Twelve-Year Medico-Legal Case Series and Comparative Analysis of Out-of-Court Versus In-Court Litigation

**DOI:** 10.1097/PTS.0000000000001525

**Published:** 2026-07-03

**Authors:** Valentina Calamano, Rosario Barranco, Fabio Comero, Giuseppe Di Pientrantonio, Isabella Caristo, Francesco Ventura

**Affiliations:** * From the Department of Legal and Forensic Medicine, Health Science Department (DISSAL), University of Genoa; † IRCCS-Ospedale Policlinico San Martino Teaching Hospital, Genoa, Italy

**Keywords:** legal medicine, hospital traumatic events, claims, public health, litigation

## Abstract

**Background:**

Falls and traumas in the hospital setting represent one of the main categories of preventable adverse events, with significant clinical, medico-legal, and economic consequences. The phenomenon particularly affects the elderly population and is amplified by demographic aging, patient clinical complexity, and the structural characteristics of health care facilities.

**Materials and Methods:**

This retrospective study analyses traumatic claims received by the Claims Management Committee of the Policlinico San Martino Hospital in Genoa between 2013 and 2024. All traumatic events occurring within the hospital and related to care or work activities were included. For each case, the event dynamics, type of injury or death, medico-legal process, and amounts paid were analyzed. Extrahospital traumas, nontraumatic claims, and cases lacking adequate documentation were excluded.

**Results:**

Out of 1796 total claims, 150 (8.35%) involved traumatic events. Cases showed a predominance of females (70.7%) with a mean age of 64 years. Structural risk was the most frequent (43.3%), followed by clinical risk (34.7%) and occupational risk (22.0%), highlighting the role of environmental hazards. Injuries were mostly fractures (79%), frequently affecting the lower limbs; cranial trauma accounted for the majority of deaths (8%). Seventy-six percent of disputes were resolved extrajudicially, while 20% proceeded to judicial settlement. The total expenditure amounted to €2,447,412.90 (≈$2,820,000), with significantly higher average costs in judicial cases.

**Conclusions:**

Hospital traumatic events have a significant clinical, medico-legal, and economic impact. Integrated preventive strategies and increased extrajudicial management are key elements to reduce traumatic events and related claims.

Trauma and patient falls during hospitalization represent a major issue in health care systems worldwide. These events lead not only to a worsening of the patient’s prognosis and clinical outcome, but also to a significant increase in the financial burden,^1^ both due to prolonged hospitalization and to compensation claims.

The incidence of falls in clinical settings varies considerably by type of health care facility. In acute care hospitals, as well as in geriatric wards and emergency departments, rates generally range between 1.34 and 8 events per 1000 patient-days.^2–6^ However, these values increase in rehabilitation facilities, where patients are actively encouraged to mobilize as an integral part of the therapeutic process. In these settings, the incidence of falls is higher, typically ranging between 3 and 16 per 1000 patient-days.^2,7,8^

According to additional studies,^9,10^ falls affect ∼50% of elderly patients hospitalized in acute care settings.

It is essential to note that about 30% of all in-hospital falls are not without consequences, resulting in injuries or harm to patients.^11,12^

According to recent studies,^13^ each year ∼1 million patients experience trauma during hospitalization each year; 25% of these sustain minor injuries, while 10% suffer severe harm. Injuries may range from minor lesions—such as lacerations, contusions, sprains, and strains—to far more serious outcomes, including head trauma, bone fractures, and, in the most unfortunate cases, death.^2^

For example, elderly patients who experience a fall in the hospital resulting in a femoral fracture have a higher likelihood of dying from complications related to the injury compared with the general population.^7,14^

Based on US studies,^13,15,16^ each traumatic event in hospitalized patients carries an additional cost of ∼35,000 dollars and has been associated with up to 11,000 deaths per year.

Geriatric patients are certainly at higher risk of falls with the potential for severe sequelae. The acute onset or exacerbation of chronic conditions exposes many patients to significant vulnerability. This often results in delirium due to unfamiliarity with the hospital environment. Physical and cognitive limitations reduce the ability to self-manage and to assess danger, factors that contribute to falls in the hospital setting. This category also includes patients who fall due to recently developed asthenia or muscle weakness, caused by fatigue or by the progression of their illness.^13^

Furthermore, the phenomenon is steadily increasing: in line with the general demographic aging, the overall annual fall rate has risen progressively by 7%, highlighting the urgency of targeted preventive strategies.^17^

Falls represent the largest category of preventable adverse events in hospitals.^17^ For this reason, it is necessary to mitigate the incidence and consequences of falls in health care facilities through the systematic implementation of evidence-based protocols and guidelines. This action is particularly critical and urgent for the elderly population, which constitutes the predominant group receiving hospital care.^18^

Alongside the well-documented issue of falls among hospitalized patients, traumatic injuries and falls involving visitors represent an additional and significant source of litigation for health care institutions. These events inevitably lead to compensation claims, especially when the incident is attributable to a hazard on the ground that was not adequately signaled or foreseeable (eg, wet floors or structural irregularities).

This maintenance and safety management problem is particularly exacerbated in hospital facilities characterized by a complex, multibuilding layout rather than a single structure. The dispersion of spaces and the extension of external areas (roads, walkways, and parking lots) inherently make preventive maintenance and continuous monitoring of ground conditions more difficult and less uniform, thereby increasing the risk of falls for external users.

This article examines traumatic incidents within the hospital and the corresponding medico-legal claims submitted to the Claims Management Committee of the Policlinico San Martino Hospital in Genoa over the period 2013 to 2024 (12 y). Epidemiological and clinical aspects, the hospital's potential liability, and related costs are evaluated.

## MATERIALS AND METHODS

The present study is a retrospective analysis of traumatic claims submitted to the Claims Management Committee (CMC) of San Martino Hospital in Genoa from January 1, 2013, to December 31, 2024. Data collection was conducted through a systematic review of health care and administrative documentation stored at the Hospital’s Legal Office, including medical records, departmental reports, investigative documents, and correspondence related to the extrajudicial and judicial phases of each dispute.

To fully understand the adopted claims-management system, it is necessary to recall the role of the Regional Claims Management Protocol, in force in the Liguria Region since 2012, which uniformly regulates the operational procedures for handling medico-legal disputes within Healthcare Organizations. This protocol is based on a self-retention insurance model, in which the Region directly assumes the economic risk of compensation claims through a dedicated fund. The claims-management process includes an initial investigative phase conducted by the Legal Office, responsible for acquiring clinical documentation and reconstructing the event. Following this phase, the hospital’s Forensic Physician prepares a technical-scientific report to assess the presence of damage, quantify it, and determine any correlation with professional or structural liability.

Once the investigation is completed, the case is submitted to the Area Claims Management Committee, composed of professionals with medico-legal, hygiene, administrative, and legal expertise. The Committee examines the documentation, evaluates the claim's legitimacy, and provides an indication of its acceptance or rejection, as well as an assessment of the appropriateness of proposing a settlement to the claimant. In more complex cases or those with greater economic impact, the file is also forwarded to the Regional Claims Committee, whose opinion is binding. The outcome of the evaluation determines the subsequent development of the dispute, which may conclude in the extrajudicial phase—through acceptance of a settlement proposal—or proceed to civil or criminal court.

For the purposes of this study, all claims classified as traumatic events occurring within the Ospedale Policlinico San Martino, or otherwise arising in direct connection with the care or work activities carried out in the hospital’s wards and services, were included. This definition encompasses, for example, accidental falls, trauma occurring during care procedures, occupational injuries involving health care personnel, and all lesions resulting from traumatic dynamics within the facility.

Conversely, trauma occurring outside the hospital setting—such as extrahospital events or incidents at home or in unrelated facilities—was excluded. Also excluded were claims not related to a traumatic event, such as nontraumatic clinical complications or therapeutic errors, as well as all requests lacking the minimum documentation necessary to reconstruct the contested episode.

For each case, the event dynamics, type of injury or death, the opinion expressed by the Claims Management Committee, and the subsequent evolution—particularly any progression to judicial proceedings and the amounts paid—were recorded. The classification of each claim into risk category was also assessed, distinguishing among clinical, structural, and occupational risks. The first refers to adverse events occurring during the patient’s care pathway; structural risk refers to damage caused by hazards related to permanent or movable structural elements within the hospital’s areas; occupational risk refers to trauma involving hospital employees.

## RESULTS

Between 2013 and 2024, the San Martino Hospital received a total of 1796 claims; among these, 150 traumatic events were selected for analysis (8.35% of all claims) (Fig. 1).

**FIGURE 1:**
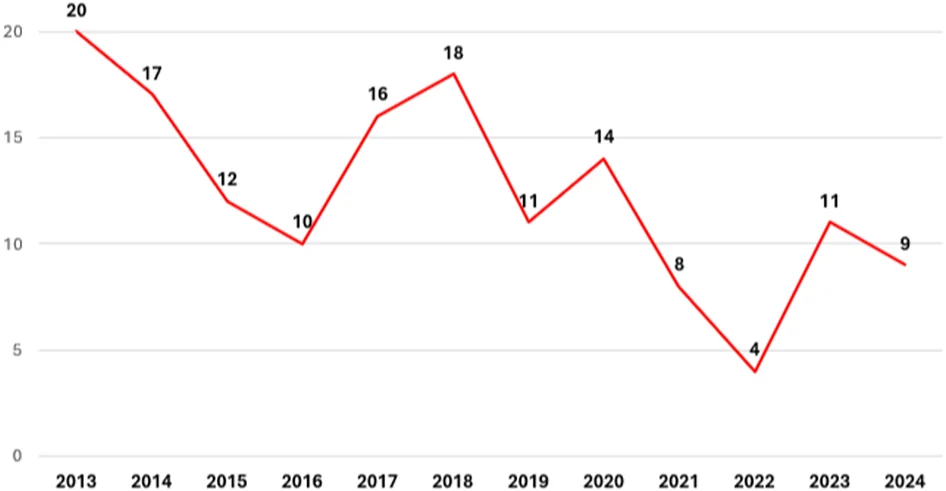
Trends in compensation claims over time.

The population of traumatic cases showed a clear female predominance: 106 cases (70.67%) versus 44 males (29.33%), with an overall mean age of 64 years.

Analyzing the type of risk, the most represented category was structural risk (65 cases, 43.33%), followed by clinical risk (52 cases, 34.67%) and occupational risk (33 cases, 22.00%). This distribution highlights how issues related to the safety of hospital environments and furnishings constitute a significant portion of the traumatic events examined.

Clinical outcomes were mostly favorable, with nonfatal injuries: 138 cases (92.00%) resulted in personal injury, while 12 cases (8.00%) had a fatal outcome. Although numerically limited, fatal cases contribute substantially to the overall medico‑legal and economic burden.

Fractures represented the most frequent type of injury, while head trauma was the leading cause of death; the detailed distribution of injury types is summarized in Table 1 and Fig. 2.

**TABLE 1:** Data on the Type of Injury and the Causes of Death for the Cases Analyzed

Injuryes types	No. Cases (%)
Bone fractures	97 (70.3)
Proximal femur fractures	17 (12.3)
Upper limb fractures (radius, ulna, humerus, scapula, and hand)	29 (21.0)
Distal lower limb fractures (ankle, foot, and metatarsal)	24 (17.4)
Rib fractures	6 (4.3)
Vertebral fractures	11 (8.0)
Multiple fractures	10 (7.3)
Sprains and ligament injuries (ankle, knee, and shoulder)	21 (15.2)
Contusions/abrasions without fracture	9 (6.5)
Dental or maxillofacial injuries	7 (5.1)
Muscle-tendon injuries	4 (2.9)
Total	138
TYPE OF FATAL INJURY	
Traumatic brain injury	9 (75)
Femoral fracture-related complications	3 (25)
Total	12

**FIGURE 2:**
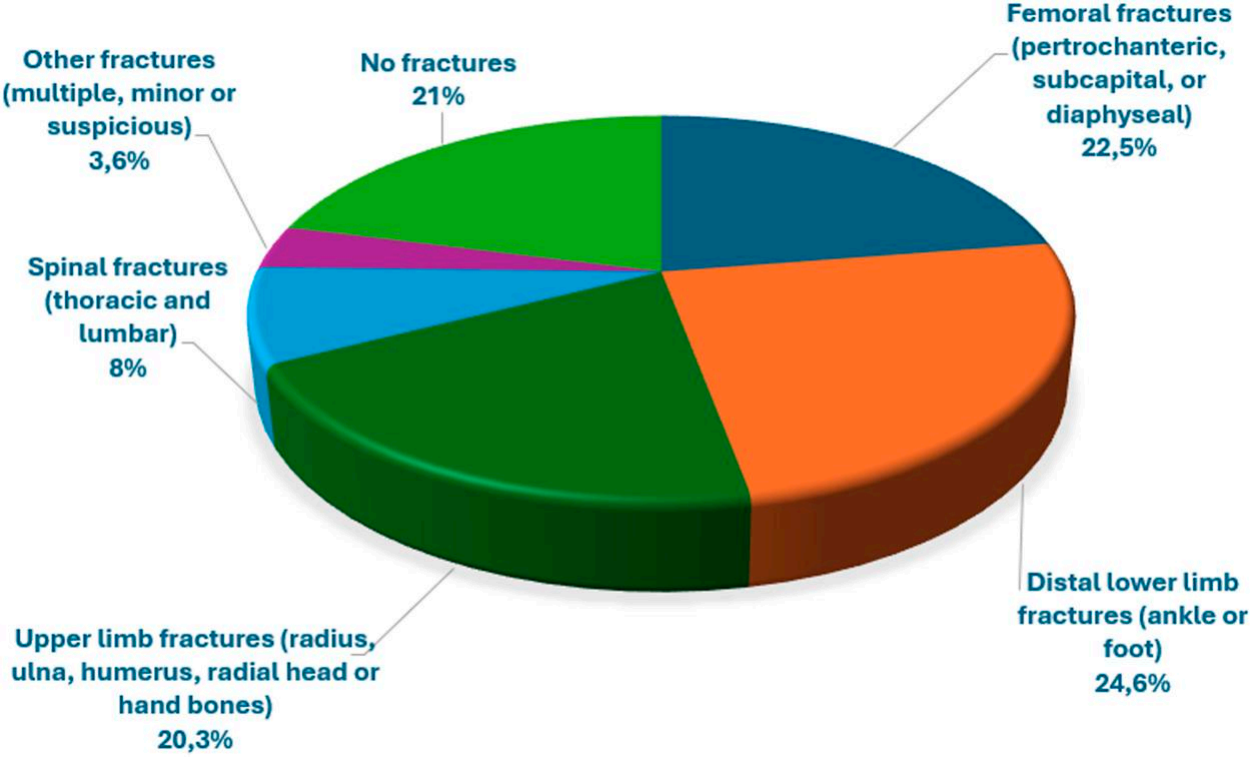
Distribution of traumatic injuries by presence or absence of fractures and type of fracture.

Regarding the procedural pathway and the evaluation by the Claims Management Committee (CMC), 92 cases (61.33%) were deemed admissible, recognizing the Institution's causation and liability.

Fifty cases (33.33%) were rejected due to lack of identifiable responsibility; 4 cases (2.67%) were referred directly to judicial proceedings, and another 4 (2.67%) remained unresolved due to insufficient or incomplete documentation.

Overall, most cases (114, 76.00%) were resolved extrajudicially, while 30 (20.00%) progressed to judicial proceedings; 6 cases remain pending.

Of the 92 claims accepted by the CMC, 90 (97.85%) have already been settled and closed, while 2 (2.15%) are still pending finalization of compensation.

Among the 50 rejected claims, 30 (60%) were closed without further action, 12 (24%) subsequently proceeded to judicial evaluation and were later compensated, while 6 (12%) remain in ongoing judicial proceedings. The total economic impact of the traumatic disputes examined to date amounts to €2,447,412.90 (≈$2,820,000), paid out in the settlement of 105 cases. The average compensation per resolved claim is therefore €23,308.69 (≈$26,860).

However, the distribution of compensation amounts shows a clear difference between extrajudicial settlements—totalling €1,500,721.30 (≈$1,729,180) across 84 cases (average €17,865.73 (≈$20,580) per case)—and judicial settlements—totalling €946,691.60 (≈$1,090,800) across 21 cases (average €45,050.55 (≈$51,900) per case). This discrepancy confirms that judicial proceedings tend to entail a significantly higher economic burden than extrajudicial resolution.

Regarding the distribution of costs by risk category, the largest share of total expenditure is attributable to clinical risk (€1,244,427.54 (≈$1,433,870), 50.85% of the total), followed by structural risk (€980,867.06 (≈$1,130,190), 40.07%) and occupational risk (€222,118.30 (≈$255,930), 9.08%). These data suggest that clinical events, although numerically less represented than some structural categories, generate a higher economic burden per case.

Finally, examining the case-closure method confirms the central role of extrajudicial management as the primary tool for containing both procedural time and costs: 76% of the traumatic disputes analyzed were resolved through settlement.

Table 2 summarizes health care litigation cases, outlining their progression into either out‑of‑court settlements or judicial proceedings, including any resulting compensation awards (Table 2).

**TABLE 2:** Summary of Health Care Litigation Cases, Detailing Their Evolution into Either Out-of-Court Settlements or Judicial Proceedings, Including Eventual Compensation Awards

Characteristics - numerical data	
Total claims reported (2013-2024)	1796
Traumatic cases reviewed	150
Share of total cases	8.35
Male	44 (29.33)
Female	106 (70.67)
Mean age	64
Structural risk	65 (43.33)
Clinical risk	52 (34.67)
Worker-related risk	33 (22.00)
Personal injuries	139 (92.67)
Fatalities	11 (7.33)
Cases approved by the CMC	92 (61.33)
Cases rejected by the CMC	50 (33.33)
Out-of-court proceedings	114 (76.00)
Judicial proceedings	30 (20.00)
Closed cases	105
Total compensation paid	€2.447.412,90 (≈$2,820,000)
Average compensation per case	€23.308,69 (≈$26,860)
Out-of-court compensation	€1.500.721,30 (≈$1,729,180)
Court-awarded compensation	€946.691,60 (≈$1,090,800)
Clinical risk compensation	€1.244.427,54 (≈$1,433,870) (50.85)
Structural risk compensation	€980.867,06 (≈$1,130,190) (40.07)
Worker-related risk compensation	€222.118,30 (≈$255,930) (9.08)

## DISCUSSION

Traumatic events occurring in hospital settings represent a significant public health issue, as they are associated with increased morbidity and mortality, as well as with a substantial medico-legal and economic impact on health care institutions.^1–3^ Falls constitute the leading cause of accidental trauma among hospitalized patients and are recognized as the largest category of preventable adverse events in hospitals. Their relevance is further amplified by the progressive aging of the population and the increasing clinical complexity of hospitalized patients.^1,2^

The present findings do not introduce entirely novel clinical evidence regarding the epidemiology of falls in health care settings; rather, they provide a comprehensive medico-legal quantification of a phenomenon that is often only anecdotally described. In particular, this study highlights the distribution of traumatic events across different risk categories and, more importantly, quantifies their economic impact by dispute resolution method. This represents a relevant contribution, as data integrating clinical, organizational, and medico-legal aspects remain limited in the current literature.

In health care organizations, risk management plays a central role in ensuring the safety of patients, health care workers, and visitors. The risks associated with traumatic events can be classified into clinical, structural, and occupational risks, each characterized by distinct causal dynamics and medico-legal and economic implications. Clinical risk includes adverse events occurring during the patient’s care pathway and encompasses, among the most frequent causes, accidental falls—often favored by advanced age, reduced functional autonomy, cognitive impairment, and polypharmacy.^7,10,16^ Occupational risk refers to trauma involving health care workers during the performance of their duties, while structural risk is related to the physical and organizational characteristics of hospital environments, such as slippery or uneven floors, unmarked level changes, inadequate lighting, and the architectural complexity of multibuilding facilities.^1,18^

In the present study, the analysis of claims revealed a predominance of events attributable to structural risk, which represents the most frequently involved category in trauma-related disputes. This finding suggests that environmental and infrastructural conditions play a central role in the genesis of traumatic events in hospital settings, affecting not only patients but also visitors and health care workers. Although less frequent, events attributable to clinical risk were characterized by more severe outcomes and a higher economic impact per case, outlining a distinct medico-legal risk profile.

International literature has extensively documented the role of individual factors in determining fall risk. Ghosh et al,^19^ in a retrospective cohort study, identified 3705 cases of falls with a mean patient age of 68.5±17.0 years, of whom 40.2% were female. They also showed that depression, the influence of alcohol or drugs, and medication use significantly increased the likelihood of falls at a higher level of severity. Therefore, their study showed a higher likelihood of severe falls in females, attributing this to gait biomechanics, greater musculoskeletal fragility, and higher mean age; the same authors also demonstrated that falls occurring during personal hygiene activities and bathroom use are associated with more severe traumatic outcomes. Fuller^20^ described how advancing age, accumulation of comorbidities, and cognitive deficits markedly increase the risk of falls and mortality, noting that falls account for up to 70% of accidental deaths in individuals aged ≥75 years.

Polypharmacy also plays a significant role: Hester et al^12^ identified the concurrent use of multiple medications as a factor associated with injurious falls in hospital settings, while Shuto et al,^21^ in a case-crossover study, reported a significant increase in fall risk associated with the use of antihypertensives, anxiolytics, hypnotics, and antiparkinsonian drugs. These findings are consistent with those of LeLaurin and LeLaurin and Shorr,^16^ who emphasized the role of adverse drug reactions in fall-risk assessment.

The results of our study show a clear predominance of females among cases of accidental falls (70.76%), with a reported mean age of 64 years, in line with what has been described in the international literature. Compared with international cohorts, our sample shows a slightly higher proportion of female subjects, which may reflect the demographic structure of the hospitalized population and the higher prevalence of frailty among older women. Moreover, the mean age in our cohort is consistent with data from acute-care and rehabilitation settings, where advanced age is a major determinant of both fall risk and injury severity.

Interestingly, the predominance of structural risk observed in our series appears more pronounced than that reported in some international studies, where clinical factors are often considered the primary drivers of in-hospital falls. This discrepancy may be explained by the inclusion of events involving not only patients but also visitors and health care workers, as well as by the architectural complexity of large multibuilding hospital facilities. These findings underline the need to consider environmental and organizational determinants as central components of fall-prevention strategies, rather than focusing exclusively on patient-related factors.

The literature also confirms that a substantial proportion of falls is associated with severe outcomes. Neuman et al^14^ demonstrated that, among residents of long-term care facilities with proximal femoral fractures, such events are associated with significantly increased mortality and worsened functional outcomes, while Fu et al^17^ identified head trauma as one of the main determinants of mortality in older adults following a fall. Data from the Centers for Disease Control and Prevention, analyzed by Bergen et al,^22^ show that falls are the leading cause of fatal and nonfatal injuries among adults aged ≥65 years in the United States, with higher incidence and severity in females.

Our study data show a clear predominance of fractures (79% of injuries), particularly involving the femur and the ankle/foot complex, while head injuries are associated with the highest mortality rate. This distribution is fully consistent with the literature, which identifies femoral fractures and traumatic brain injuries as the main determinants of greater clinical severity and mortality.

Beyond clinical consequences, traumatic events in hospital settings also entail substantial economic costs. Dykes et al^15^ demonstrated that both injurious and noninjurious falls lead to an increase in health care costs exceeding 35,000 dollars per event, and that the implementation of evidence-based prevention programs is associated with a significant reduction in economic burden. Fields et al^23^ highlighted the high cost of imaging studies related to falls, particularly head CT scans, while Dunne et al^24^ showed that in-hospital falls significantly increase length of stay regardless of injury severity.

An additional study conducted by Lin et al^25^ involved a total of 160 hospitalized patients (80 who had experienced falls and 80 who had not, in the control group). The results show that the risk factors for in-hospital falls included the absence of family accompaniment and the use of more than 3 medications. The findings also indicated that medical costs increased with the severity of injuries, with an average medical cost of 18,257 Taiwanese dollars for patients with third-degree injuries.

Consistent with this evidence, the results of the present study show that the average claim cost varies significantly by risk type and dispute-resolution method. In particular, compensation claims related to clinical-risk events and those resolved through judicial proceedings were associated with a substantially higher economic burden than those related to structural-risk events and those resolved extrajudicially. This finding confirms that the severity of clinical outcomes and the complexity of the care and legal processes are key determinants of the overall economic impact. This interpretative framework is further reinforced by the observation that, despite the higher frequency of structural-risk events, clinical-risk events are associated with a higher economic impact per case. This is particularly relevant from a medico-legal perspective, as it suggests that the severity of clinical outcomes and the complexity of care significantly influence the magnitude of compensation claims. Moreover, the observed difference between the costs of extrajudicially resolved disputes and those that progress to judicial proceedings confirms that the legal process itself is an additional factor contributing to an increased economic burden. From a medico-legal and organizational perspective, this finding has important implications. The significantly higher costs associated with judicial proceedings suggest that early assessment and structured extrajudicial management of claims may represent an effective strategy not only for reducing litigation time but also for containing overall health care expenditure. In this context, the role of multidisciplinary claims management committees appears crucial in promoting timely decision-making and facilitating settlement when appropriate. These results are consistent with observations from other health care systems, where alternative dispute resolution mechanisms have been associated with lower legal costs and improved efficiency in claim management. Therefore, strengthening extrajudicial pathways may represent a key element of risk management policies.

At the national level, comparison with data from the MedMal report^26^ allows the results of the present study to be contextualized within the broader Italian landscape of health care litigation. Although the distribution of dispute-resolution modalities is broadly consistent with national data, our sample shows a lower proportion of claims closed without further action and a relatively higher proportion of traumatic events with injurious outcomes. This aspect reinforces the interpretation of the observed economic results, as the lower number of archived claims and the higher frequency of injurious events—more often associated with compensation—help explain the elevated average compensation observed.

Overall, available data indicate that the most effective prevention strategies are multifactorial and must consider the different risk categories. Structural interventions to improve environmental safety, systematic fall-risk assessment programs, medication-therapy reviews, and health care worker training are essential tools for reducing traumatic events and the resulting litigation.^18,27^

This study has several limitations, primarily related to its retrospective, single-center design and to the selection of cases based on claims subject to litigation, which may have favored the inclusion of traumatic events with greater clinical severity and medico-legal relevance, thereby limiting the generalizability of the results to other care settings. Future studies-ideally prospective and multicentre that integrate clinical, organizational, and medico-legal data could allow for a more comprehensive assessment of the phenomenon and improve the representativeness of findings, contributing to the identification of more effective preventive and management strategies.
